# The Prognostic Value of Proclarix in Prostate Cancer Patients Under Active Surveillance: Predicting Transition to Active Treatment and Disease Progression in a Danish Cohort

**DOI:** 10.3390/cancers18091348

**Published:** 2026-04-23

**Authors:** Alcibiade Athanasiou, Torben F. Hansen, Jonna S. Madsen, Mads H. Poulsen, Mike Allan Mortensen, Gitte E. Kissow, Louise F. Øbro, Palle J. Osther, Ralph Schiess, Ahmed H. Zedan

**Affiliations:** 1 Proteomedix AG, 8952 Schlieren, Switzerland; 2 Department of Oncology, Lillebaelt Hospital, University Hospital of Southern Denmark, 7100 Vejle, Denmark; 3 Department of Regional Health Research, University of Southern Denmark, 5230 Odense, Denmark; 4 Department of Biochemistry and Immunology, Lillebaelt Hospital, University Hospital of Southern Denmark, 7100 Vejle, Denmark; 5 Department of Urology, Esbjerg and Grindsted Hospital, University Hospital of Southern Denmark, 6700 Esbjerg, Denmark; 6 Department of Urology, Odense University Hospital of Southern Denmark, 5000 Odense, Denmark; 7 Urological Research Center, Department of Urology, Lillebaelt Hospital, University Hospital of Southern Denmark, 7100 Vejle, Denmark

**Keywords:** active surveillance, biomarker, cathepsin D, PerPros biobank, Proclarix, prognosis, prostate cancer, prostate-specific antigen, risk score, thrombospondin-1

## Abstract

Background and Objective: Active surveillance (AS) describes the active monitoring of men with low- to intermediate-risk prostate cancer (PCa), before active management (AM) is needed due to disease progression. A substantial proportion of patients require a transition to AM within a few years of diagnosis. Proclarix is a blood-based diagnostic test that predicts clinically significant PCa (csPCa) and the Proclarix risk score has been shown to correlate with tumor aggressiveness. This study aimed to assess whether Proclarix can predict the likelihood of transition from AS to AM and to compare it to PSA density (PSAD). Methods: We retrospectively evaluated the Proclarix risk scores in serum samples from a Danish cohort of 132 men recruited from the PerPros prostate biobank. Most participants had low- to intermediate-risk PCa and were considered eligible for AS at diagnosis. Blood samples were collected before the initial biopsies, and clinical follow-up data were available for every patient for a minimum of 3 and up to 9.5 years. The primary endpoint was the ability of the Proclarix risk score to predict the transition from AS to AM. The secondary endpoint was to assess whether Proclarix could identify patients at risk of progression to csPCa. For both endpoints, PSA density was also included in the analysis for comparison. Results: Overall, 48 of 132 men (36%) transitioned from AS to AM during follow-up. A baseline Proclarix risk score of ≥50% was associated with a 79% estimated cumulative probability of switching to AM (HR = 4.4, 95% CI: 2.3–8.3, *p* < 0.001), compared to the 58% (HR = 3.1, 95% CI: 1.7–5.7, *p* < 0.001) for PSAD At the 5-year follow-up, 82% of men with a Proclarix score ≥ 50% and 57% with PSAD ≥ 0.15 ng/mL/cm^3^ had progressed to AM. Additionally, 67% and 54% of men showed progression to csPCa with, respectively, Proclarix and PSAD at the confirmatory biopsy. In contrast, among men with a Proclarix score < 50%, only 28% progressed to AM and 32% to csPCa, whereas for PSAD < 0.15 ng/mL/cm^3^, 17% transitioned to AM and 23% progressed to csPCa. Conclusions: The Proclarix risk score may support clinical decision-making in AS by identifying patients at higher risk of progression and informing follow-up intensity. However, the results should be confirmed in a larger prospective study.

## Simple Summary

Active surveillance (AS) is the recommended management approach for patients diagnosed with low/intermediate/risk prostate cancer. However, 40% of patients under AS will require active management (AM) within the first 5 years of observation. In this study, Proclarix risk score was investigated as a prognostic tool for both transition from AS to AM and progression of biopsy Grade Group (GG), using baseline serum samples from a Danish cohort of 132 men under AS. At the 5-year follow-up, 82% of men with a baseline Proclarix score > 50% had progressed to AM, and 67% showed GG progression at the confirmatory biopsy. The Proclarix risk score may assist in tailoring the monitoring program for prostate cancer patients undergoing AS.

## 1. Introduction

Active surveillance (AS) is widely recommended as a preferred management strategy for men diagnosed with low-risk or favorable intermediate-risk prostate cancer (PCa) [1,2]. Since its introduction in the 1990s, AS has aimed to delay or avoid altogether active management (AM), such as radical prostatectomy or radiotherapy, thereby reducing treatment-related morbidity while safely monitoring men with clinically insignificant PCa (ciPCa) [3]. Nevertheless, disease reclassification over time is common, and 20–38% of men transition to AM within five years after the diagnostic biopsy [4,5].

Current AS monitoring is largely based on a combination of clinical assessment, serial serum prostate-specific antigen (PSA) measurements, and multiparametric magnetic resonance imaging (mpMRI), often complemented by repeat biopsies. However, the limited consensus on the optimal intensity and frequency of follow-up has contributed to heterogenous implementation and suboptimal uptake of AS worldwide. Despite broad guideline support, a considerable proportion of men (around 40%) with ciPCa still undergo potentially unnecessary definitive treatment [6].

To refine patient selection and tailored follow-up intensity, PSA density has been included as selection criterion using a cut-off of 0.15 ng/mL/cm^3^ [1]. Additionally, multiple other approaches have been proposed, including clinical risk factors [7,8] risk-stratification nomograms [9], and both tissue- and blood-based biomarkers [10,11,12]. Yet, these tools have only modestly improved the ability to balance the competing risks of overtreatment and delayed intervention. There is a clear need for better strategies that identify men who can be safely monitored with less intensive follow-up and those at higher risk of progression who may benefit from earlier treatment.

Proclarix is a CE-IVD certified blood test developed to support early detection of csPCa. It provides an individualized risk score derived from the tumor-associated biomarkers cathepsin D (CTSD) and Thrombospondin 1 (THBS1), combined with total PSA, percent free PSA, and age. Multiple studies have shown that Proclarix predicts clinically significant PCa (csPCa) in biopsy-naïve men with suspected disease, with a reported negative predictive value of approximately 95%, enabling the avoidance of unnecessary biopsies [13,14,15,16,17]. In addition, the Proclarix risk score has been shown to correlate with tumor aggressiveness [18].

On this basis, we hypothesized that Proclarix could also be useful in the AS setting to estimate the likelihood of the transition to AM and to identify men at increased risk of progression from ciPCa to csPCa.

## 2. Materials and Methods

### 2.1. Study Design and Population

Proclarix risk scores were measured retrospectively in baseline serum samples from 132 men diagnosed with PCa who were managed with AS according to the Danish guidelines [19]. Patients had been prospectively enrolled in the PerPros (Personalized Management of Prostate Cancer) biobank at the Department of Urology, Lillebaelt Hospital, University Hospital of Southern Denmark, Vejle, Denmark (jr.nr: 18/11174).

**Figure 1:**
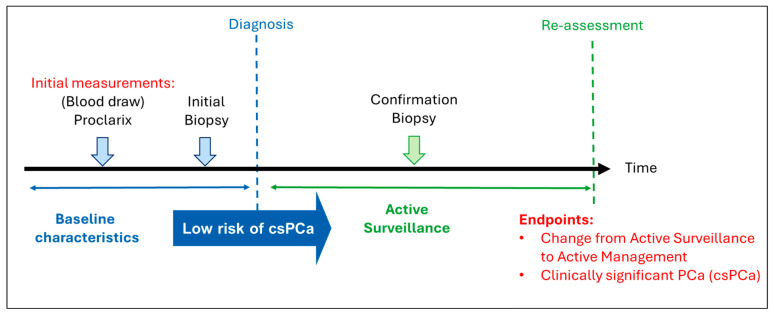
Schematic overview of the study design. Proclarix was measured retrospectively in baseline blood samples collected prospectively before biopsy. Endpoints were transition from AS to AM and progression to csPCa during follow-up.

Participants were referred to the Department of Urology at Vejle or Esbjerg Hospital for further evaluation of suspected PCa, most commonly due to elevated PSA levels. Decisions regarding the initiation of AS and the subsequent transition to AM during follow-up were made predominantly at the Department of Urology, Odense University Hospital of Southern Denmark, Odense, Denmark. All participants provided written informed consent. The study workflow is summarized in Figure 1.

### 2.2. Proclarix and PSAD Assessment

Baseline blood samples were obtained prior to the initial prostate biopsy. To obtain serum, blood samples were allowed to clot for a timeframe of 30–60 min before being centrifuged at 2000× *g* for 10 min at room temperature. Serum was aliquoted and stored at −80 °C until analysis.

Proclarix is a blood-based biomarker test that combines age, tPSA, %fPSA, and the two tumor markers CTSD and THBS1 in an algorithm generating a 0–100% risk score, originally designed to predict csPCa [14]. Proclarix measurements were performed blinded to all clinical information and biopsy results.

For all samples, serum tPSA and %fPSA were remeasured using the Roche Cobas immunoassay system (Roche Diagnostics, Rotkreuz, Switzerland), THBS1 and CTSD were quantified using the CE-marked Proclarix kit (Proteomedix, Schlieren, Switzerland) [20].

PSAD was calculated as tPSA divided by the prostate volume, which was determined by transrectal ultrasound (TRUS)/mpMRI of the prostate.

### 2.3. Study Outcomes

The primary endpoint was the ability of the baseline Proclarix risk score to predict a transition in management from AS to AM (surgery, radiotherapy or androgen deprivation therapy alone) within 3 years and 5 years after the initial biopsy.

The secondary endpoint was the association between the baseline Proclarix risk score and the risk of progression from the initial biopsy Grade Group (GG) to csPCa on subsequent biopsies within 3- and 5-year follow-up periods. In this study csPCa was defined as GG ≥ 2, according to the International Society of Urological Pathology criteria [21]. Follow-up was censored on 2 May 2025. For both endpoints, PSA density was also included in the analysis for comparison.

### 2.4. Statistical Analysis

Group comparisons (progression vs. no progression) were performed using a *t*-test. Time-to-event analyses were conducted using Kaplan–Meier methods to compare cumulative event probabilities between low- and high-risk groups, and Cox proportional hazards models were fitted to estimate hazard ratios (HRs) with 95% confidence intervals and Wald *p*-values. The proportional hazards assumption of the Cox model was assessed using the Schoenfeld’s approach [22,23].

For analyses of both the transition from AS to AM and the progression to csPCa, patients were stratified into low-risk and high-risk groups using a PSAD cut-off of 0.15 ng/mL/cm^3^, as proposed in the guidelines [1] and a Proclarix risk score cut-off of 50%. This threshold was selected to yield a high-risk group with a cumulative probability > 75% at 5 years following the initial biopsy.

Clinical performance of Proclarix and PSAD was evaluated, and comparisons of sensitivity and specificity were performed using McNemar’s test [24]. Differences in negative and positive predictive values (NPV, PPV) were assessed using the method of Moskowitz and Pepe [25]. Comparisons of the area under the ROC curve (AUC) were performed according to DeLong et al. [26], using the Sun and Xu algorithm [27].

Analyses were performed in R (version 4.0.2, the R Foundation for Statistical Computing, Vienna, Austria), using the survival, DTComPair and survminer packages, in addition to basic R functions. A two-sided *p*-value of <0.05 was considered statistically significant.

## 3. Results

### 3.1. Baseline Characteristics

Between October 2015 and May 2022, 132 men diagnosed with localized PCa and managed with AS were eligible for inclusion. Initial prostate biopsies were performed as transrectal ultrasound (TRUS)-guided systematic biopsies in 105/132 (80%) patients and as mpMRI-guided biopsies in 27/132 (20%) patients. During follow-up, 98/132 (74%) patients underwent at least one confirmatory biopsy. Among these, 72/98 (73%) had at least one confirmatory biopsy performed with mpMRI guidance. Most of the patients had a baseline tPSA < 10 ng/mL (105/132, 80%), while 7/132 (5%) had a tPSA > 20 ng/mL. At the initial biopsy, most patients were classified as Grade Group (GG) 1 (107/132, 81%), 23/132 (17%) were GG2, and 2/132 (2%) were >GG2 (one GG3, one GG5). Over the available follow-up period, 48/132 (36%) patients transitioned from AS to AM, mainly due to GG progression at confirmatory biopsy according to the national Danish guidelines. The median follow-up after diagnosis was 6.3 years (range: 3.0–9.4 years). Baseline demographics and clinical characteristics are summarized in Table 1.

### 3.2. Change to Active Management

**Figure 2:**
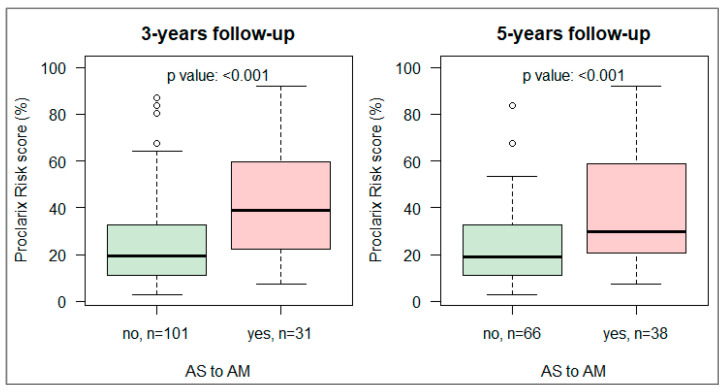
Boxplot of baseline Proclarix risk score by transition from AS to AM. (**Left**) At least 3 years of follow-up. (**Right**) At least 5 years of follow-up.

At three years of follow up, 31/132 (23%) patients had transitioned from AS to AM, and at five years of follow up, 38/104 (37%) patients (Figure 2). The baseline Proclarix risk score was significantly associated with the transition from AS to AM in both the 3-year and 5-year follow-up cohorts (*p* < 0.001, Figure 2).

**Figure 3:**
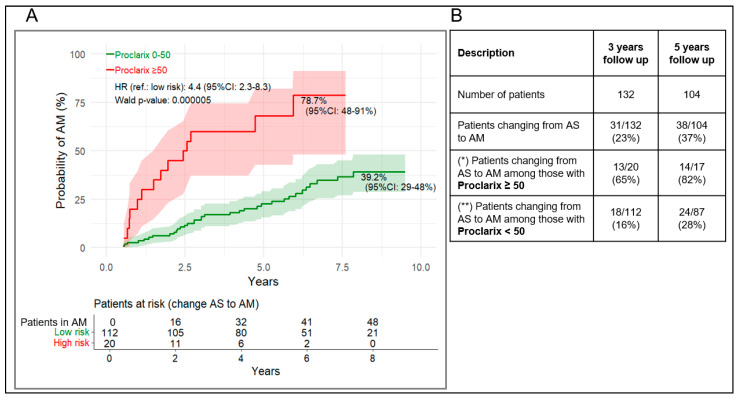
(**A**) Kaplan–Meier curves for cumulative probability of transition from AS to AM stratified by Proclarix risk score ≥ 50% vs. <50%. (**B**) Proportion transitioning after at least 3 and 5 years. (*) as for PPV in the clinical performance analysis, (**) as for 100-NPV in the clinical performance analysis. 95% CI of (*) and (**) are listed in Table 2.

**Figure 4:**
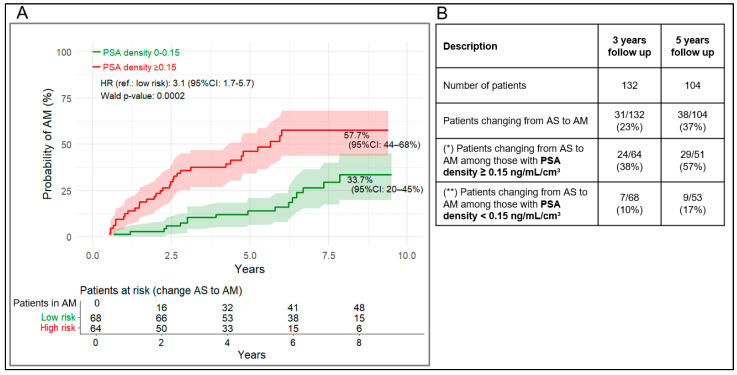
(**A**) Kaplan–Meier curves for cumulative probability of transition from AS to AM stratified by PSAD ≥ 0.15 vs. <0.15 ng/mL/cm^3^. (**B**) Proportion transitioning after at least 3 and 5 years. (*) same as clinical performance PPV, as for PPV in the clinical performance analysis, (**) as for 100-NPV in the clinical performance analysis. 95% CI of (*) and (**) are listed in Table 2.

Using a 50% Proclarix risk score cut-off or a 0.15 ng/mL/cm^3^ PSAD cut-off, patients with scores above the cut-off showed an estimated 78.7% (95% CI: 48–91%) and 57.7% (95% CI: 44–68%) cumulative risk of transitioning from AS to AM, respectively, during follow-up (Figure 3A and Figure 4A). A value above the Proclarix’ and PSAD’ cut-off was associated with a markedly four- and three-fold increased risk of transition to AM, respectively, compared with values below the cut-off (HR = 4.4, 95% CI: 2.3–8.3, *p* < 0.001, respectively HR = 31, 95% CI: 1.7–5.7, *p* < 0.001)). Schoenfeld residual tests (Figure S1) showed no significant *p*-values for Proclarix, while PSAD showed a slight violation after 5 years (*p* = 0.024) that was not present over the full period (*p* = 0.29), indicating that the proportional hazards assumption was overall not violated.

In the 3- and 5-year follow-up cohorts, a PPV of 65% (95% CI: 44–86%) and 82% (95% CI: 64–100%) were observed for patients with a Proclarix risk score ≥ 50% who transitioned to AM, compared with 16% (100-NPV value, 95% CI: 9–23%) and 28% (100-NPV value, 95% CI: 18–37%) among those with a Proclarix risk score < 50%, respectively (Figure 3B, Table 2).

Similarly, for PSAD, the observed PPVs at 3 and 5 years were 38% (95% CI: 26–50%) and 57% (95% CI: 44–75%), respectively. In contrast, among patients with a PSAD < 0.15 ng/mL/cm^3^, the corresponding rates were 10% (100-NPV, 95% CI: 3–17%) and 17% (100 − NPV, 95% CI: 7–27%) (Figure 4B, Table 2).

Table 2 shows that, despite similar AUC values for Proclarix and PSAD (*p* > 0.300), Proclarix demonstrated at least 25% higher PPV and specificity than PSAD, with differences consistently reaching statistical significance (*p* < 0.05). In contrast, PSAD showed a significantly higher sensitivity, by at least 30% compared to Proclarix (*p* < 0.05). NPV was only slightly higher for PSAD and reached statistical significance at 5 years (*p* = 0.018), but not at 3 years (*p* = 0.08).

### 3.3. Progression to Clinically Significant Prostate Cancer

At the initial biopsy, 25/132 (19%) patients were diagnosed with csPCa (GG ≥ 2). These patients were excluded from the analyses assessing the progression from GG1 to csPCa during follow-up. Consequently, 107 patients were included in the 3-year progression analysis and 83 patients in the 5-year analysis.

**Figure 5:**
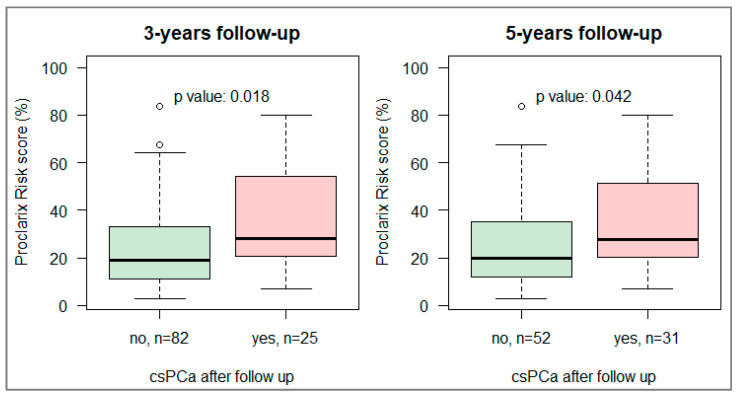
Boxplot of baseline Proclarix risk score by progression to csPCa at follow-up biopsy. (**Left**) At least 3 years of follow-up. (**Right**) At least 5 years of follow-up.

During the follow-up, 25/107 (23%) and 31/83 (37%) patients progressed to csPCa after at least 3- and 5-year follow-up, respectively (Figure 5). Baseline Proclarix risk scores were significantly associated with the progression to csPCa in both analyses (*p* = 0.018 at 3 years and *p* = 0.042 at 5 years), Figure 5.

**Figure 6:**
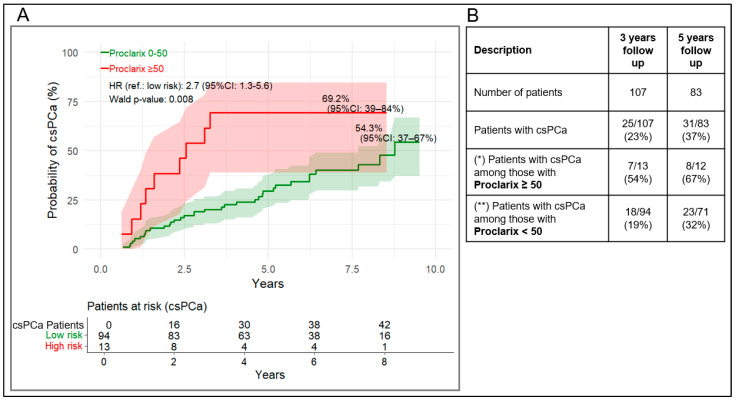
(**A**) Kaplan–Meier curves for cumulative probability of progression to csPCa stratified by Proclarix risk score ≥ 50% vs. <50%. (**B**) Proportion transitioning after at least 3- and 5-years follow-up. (*) as for PPV in the clinical performance analysis, (**) as for 100-NPV in the clinical performance analysis. 95% CI of (*) and (**) are listed in Table 3.

**Figure 7:**
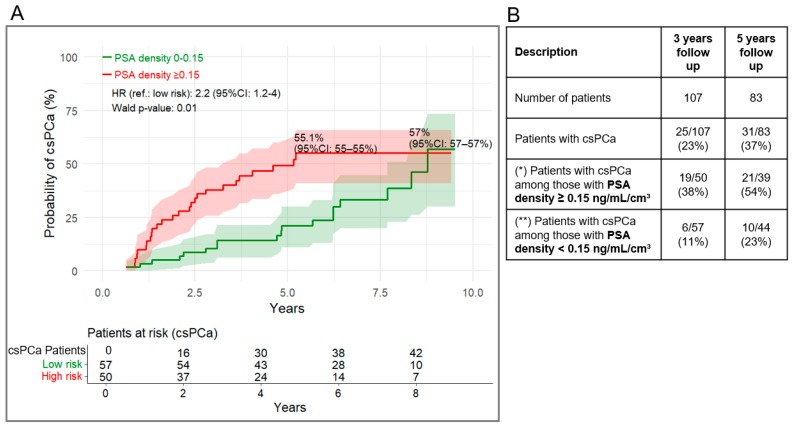
(**A**) Kaplan–Meier curves for cumulative probability of progression to csPCa stratified by PSAD ≥ 0.15 vs. <0.15 ng/mL/cm^3^. (**B**) Proportion transitioning after at least 3- and 5-years follow-up. (*) as for PPV in the clinical performance analysis, (**) as for 100-NPV in the clinical performance analysis. 95% CI of (*) and (**) are listed in Table 3.

Using the 50% cut-off, patients with a Proclarix risk score above the cut-off had an estimated 69.2% (95% CI: 39–84%) probability of progression to csPCa during follow-up (Figure 6A). For PSAD, using a cut-off of 0.15 ng/mL/cm^3^, the corresponding probability was 55.1% (95% CI: 37–67%) (Figure 7A). Schoenfeld residual tests (Figure S1) showed no significant *p*-values for Proclarix, while PSAD showed no violation after 5 years (*p* = 0.47) but a significant result over the full period (p < 0.001), suggesting a possible time-dependent effect for PSAD.

Values above the cut-off for Proclarix and PSAD were associated with a 2.7-fold(95% CI: 1.3–5.6, *p* = 0.008), and 2.2-fold (95% CI: 1.2–4.0, *p* = 0.01) higher risk of progression, respectively, compared with values below the cut-off. In the 3- and 5-year follow-up cohorts, 54% (PPV, 95% CI: 27–81%) and 67% (PPV, 95% CI: 40–93%) of patients with a Proclarix risk score ≥ 50% progressed to csPCa, whereas 18% (100-NPV, 95% CI: 73–89%) and 32% (100-NPV, 95% CI: 57–78%) of patients with a Proclarix risk score ≤ 50% progressed, respectively (Figure 6B, Table 3). Similarly, for PSAD, the PPVs at 3 and 5 years were 39% (95% CI: 25–52%) and 54% (95% CI: 39–71%), respectively (Figure 7B, Table 3).

Similarly to Table 2, Table 3 shows that AUC values for Proclarix and PSAD were not significantly different (*p* > 0.1). Proclarix and PSAD also demonstrated comparable PPV and NPV, with all comparisons showing *p* > 0.05, except for the NPV of PSAD, which was slightly but significantly higher than that of Proclarix (*p* = 0.038). Additionally, Proclarix demonstrated a significantly higher PPV and specificity than PSAD (*p* < 0.05). In contrast, PSAD showed a significantly higher sensitivity compared to Proclarix (*p* < 0.05).

## 4. Discussion

This study provides initial evidence that the Proclarix risk score may have clinical value as a prognostic tool in men managed with AS. Specifically, a high Proclarix risk score at baseline was associated with both (i) a high probability of the transition from AS to AM and (ii) a higher risk of the progression to csPCa during follow-up.

Proclarix was developed to identify csPCa prior to the prostate biopsy, but evidence suggests that the risk score provides relevant information about tumor aggressiveness. Previous work has reported an association between the Proclarix risk score and the baseline diagnostic GG, clinical tumor stage (cT), adverse pathology, and biochemical recurrence after primary treatment [18]. These observations formed the rationale for evaluating whether Proclarix may also inform outcomes that are central to AS programs, namely, the need for treatment escalation and the prediction of histologic progression.

In our cohort, the Proclarix risk score at baseline showed a statistically significant association with both endpoints. Patients with a Proclarix risk score ≥ 50% had a higher cumulative probability of transitioning to AM and a higher risk of progression to csPCa compared with those with a risk score < 50%. At 5-year follow-up, 82% of patients with a Proclarix risk score ≥ 50% transitioned to AM and 67% progressed to csPCa, whereas the corresponding proportions in the group with a risk score < 50% were 28% and 32%, respectively. In comparison, PSAD with a cut-off of 0.15 ng/mL/cm^3^ showed a significantly lower risk of transition to AM at 3 and 5 years (38%, *p* = 0.003 and 58%, *p* = 0.032, respectively). The risk of progression to csPCa was also lower than that observed with Proclarix, although this difference was not statistically significant (*p* > 0.05). Together, these findings suggest that Proclarix could potentially enable a more individualized surveillance strategy, helping to identify men who may benefit from closer monitoring and earlier confirmatory assessment, while allowing less intensive follow-up in men at lower risk.

The 50% Proclarix cut-off was selected to ensure that patients in the high-risk group would have a cumulative probability of >75% at 5 years following the initial biopsy. This approach reflects a “rule-in” test, intended to support decisions on whether a patient should transition to AM. Using this cut-off, a high specificity of at least 92% was achieved at 3 and 5 years after diagnosis for both endpoints (AM and csPCa). In other words, nearly all cases were correctly identified. Specificity was significantly higher (*p* < 0.001) for Proclarix compared to the PSAD with a 0.15 ng/mL/cm^3^ cut-off, which ranged between 61% and 68%.

The study population was selected according to the EAU risk stratification [1], reflecting a real-world AS setting. Most men were classified as low risk or favorable intermediate risk, and nearly all had localized disease (cT ≤ cT2c). A small number of patients had features that would be considered higher risk (e.g., a PSA > 20 ng/mL, ≥GG3 at baseline, or cT3), yet were managed with AS based on shared decision-making and patient preference. We intentionally retained these patients to reflect routine clinical practice, acknowledging that this choice may introduce heterogeneity but improves the clinical relevance of the cohort.

Appropriate monitoring is crucial, because a substantial proportion of men on AS will undergo treatment escalation within five years after diagnosis [5]. In our study, 37% of men transitioned from AS to AM within 5 years, which aligns with discontinuation rates reported in large AS cohorts such as PRIAS (where approximately one-third discontinue due to protocol-based reclassification at 5 years) [2,5,28]. This concordance supports the external plausibility of our cohort and our endpoint definitions. We chose 5-year follow-up as a clinically meaningful time horizon: it is sufficiently long to capture early reclassification events consistent with pre-existing aggressive biology, while beyond this timeframe it becomes increasingly difficult to disentangle baseline aggressiveness from de novo progression and to determine whether curative treatment remains appropriate as age and comorbidity accumulate.

Current guideline-based follow-up strategies rely primarily on PSA, digital rectal examination, and MRI, with confirmatory biopsies performed depending on baseline risk and subsequent findings [1]. Importantly, PSA kinetics or MRI changes (while still organ-confined) generally should not trigger definitive treatment without histologic confirmation, underscoring the need for tools that can help determine who requires earlier and more intensive confirmatory evaluation [29,30].

Several tissue-based genomic tests (e.g., Oncotype Dx, Prolaris, Decipher, and ProMark) have been proposed to refine the risk stratification in AS [31], but their broader uptake is limited by the need for biopsy tissue, cost, and limited comparative evidence. Consequently, there remains an unmet medical need for non-invasive, reproducible biomarkers that can help to personalize the AS intensity.

In this context, serum-based biomarkers have gained attention, like PSAD, which was used as a comparator in this study. PSAD is promising [8], yet besides the recommended 0.15 ng/mL/cm^3^, other published cut-offs vary widely (approximately 0.08 to 0.2) [32,33,34,35], limiting consistency across cohorts and creating uncertainty regarding generalizability. The 4Kscore has also been associated with csPCa outcomes in AS populations [36,37]. Notably, Hougen et al. [37] reported that a 4KScore cut-off of 20%, stratified cumulative per-protocol progression rates over 36 months. While cross-study comparisons must be interpreted cautiously due to differences in cohorts, endpoints, and follow-up schedules, our results suggest that Proclarix may provide a more meaningful risk separation for both treatment escalation and histologic progression.

Strengths of this study include recruitment through the PerPros biobank framework, blinded Proclarix assessment, and the long-term follow-up. Key limitations are the sample size (only 48 transitions to AM), the retrospective design, and changes in the biopsy technique over time (limited MRI-targeted biopsies at baseline but more frequent during confirmatory biopsies), which may contribute to grade migration [38].

A notable limitation of this study is that the Cox proportional hazards model was not adjusted for additional covariates, as the aim of this study was to assess the prognostic performance of Proclarix and PSAD as independent biomarkers. Consequently, only a correlation between the Proclarix risk score and the transition to AM or progression to csPCa may be shown, and this may not be independent of clinical variables. The potential predictive usefulness of Proclarix beyond recognized clinical characteristics warrants investigation in future studies with larger, better-powered cohorts. Larger, prospective studies are needed to validate these initial results and the proposed cut-off, and to assess the potential added value of Proclarix beyond established clinical variables and MRI. Ideally, future work should include longitudinal Proclarix measurements to evaluate whether changes over time further improve risk prediction and guide the surveillance intensity.

## 5. Conclusions

In this exploratory study, a baseline Proclarix risk score measured prior to the initial prostate biopsy was associated with adverse pathological findings at the confirmatory biopsy and with a higher likelihood of the transition from AS to AM. These results support the potential role of Proclarix as a non-invasive prognostic tool to aid the risk stratification and guide individualized monitoring intensity in men undergoing AS. Prospective studies in larger cohorts are warranted to confirm these findings and to define clinically meaningful cut-offs and integration with established clinical and imaging parameters.

## Figures and Tables

**Table 1:** Patient demographics.

Parameter	Description	Value
Total patients, n (%)	-	132 (100%)
Age, median (min–max)	-	66 (50–81)
tPSA, n (%)	<10 ng/mL	105 (80%)
	10–20 ng/mL	20 (15%)
	>20 ng/mL	7 (5%)
clinical stage, n (%)	cT1c	103 (78%)
	cT2	5 (4%)
	cT2a	13 (10%)
	cT2b	8 (6%)
	cT2c	3 (2%)
	cT3	1 (1%)
GG at first biopsy, n (%)	GG1	107 (81%)
	GG2	23 (17%)
	>GG2	2 (2%)
GG after max. years of follow up, n (%)	GG1	63 (48%)
	GG2	54 (41%)
	>GG2	15 (11%)
EAU risk groups, n (%)	Low	81 (61%)
	Intermediate favorable	39 (30%)
	Intermediate unfavorable	6 (4.5%)
	High	6 (4.5%)
Management change after max years of follow up, n (%)	AS to AM	48 (36%)
Number of patients with follow-up	3 years	132 (100%)
	5 years	95 (72%)
	7 years	83 (63%)
	9 years	57 (53%)

AM: active management, AS: active surveillance, GG: Grade Group, tPSA: Total prostate-specific antigen.

**Table 2:** Clinical performance of Proclarix compared to PSAD at 3 and 5 years for predicting transition from AS to AM.

Cohort Description	Transition from AS to AM 3 Years Follow-Up, n = 132			Transition from AS to AM 5 Years Follow-Up, n = 104		
Test	Proclarix	PSA Density	*p*-Value	Proclarix	PSA Density	*p*-Value
Cut-off	50	0.15	-	50	0.15	-
AUC (95% CI)	0.721 (0.616–0.825)	0.759 (0.661–0.858)	0.486	0.733 (0.633–0.833)	0.789 (0.698–0.880)	0.304
Sen., % (95% CI)	42 (25–59)	77 (63–92)	0.001	37 (22–52)	76 (63–90)	<0.001
Spe., % (95% CI)	93 (88–98)	61 (52–71)	<0.001	95 (90–100)	68 (57–79)	<0.001
NPV, % (95% CI)	84 (77–91)	90 (83–97)	0.08	72 (63–82)	83 (73–93)	0.018
PPV, % (95% CI)	65 (44–86)	38 (26–50)	0.003	82 (64–100)	58 (44–72)	0.032

AS: active surveillance, AM: active management, AUC: area under the curve, Spe.: specificity, Sen.: sensitivity, NPV: negative predicted value, PPV: positive predicted value.

**Table 3:** Clinical performance of Proclarix compared to PSAD at 3 and 5 years for predicting progression to csPCa.

Cohort Description	Progression from csPCa 3 Years Follow-Up, n = 107			Progression from csPCa 5 Years Follow-Up, n = 83		
Test	Proclarix	PSA Density	*p*-Value	Proclarix	PSA Density	*p*-Value
Cut-off	50	0.15	-	50	0.15	-
AUC (95% CI)	0.668 (0.547–0.790)	0.754 (0.654–0.854)	0.190	0.638 (0.512–0.763)	0.731 (0.620–0.842)	0.184
Sen., % (95% CI)	28 (10–46)	76 (59–93)	0.001	26 (10–41)	68 (51–84)	0.001
Spe., % (95% CI)	93 (87–98)	63 (53–74)	<0.001	92 (85–100)	67 (55–80)	0.001
NPV, % (95% CI)	82 (73–89)	89 (82–97)	0.032	68 (57–78)	77 (66–90)	0.054
PPV, % (95% CI)	54 (27–81)	38 (25–52)	0.204	67 (40–93)	54 (39–71)	0.38

csPCa: clinically significant prostate cancer, AUC: area under the curve, Spe.: specificity, Sen.: sensitivity, NPV: negative predicted value, PPV: positive predicted value.

## Data Availability

The datasets used and/or analyzed during the current study are available from the corresponding author upon reasonable request.

## Supplementary Materials

The following supporting information can be downloaded at: https://www.mdpi.com/article/10.3390/cancers18091348/s1, Figure S1: Schoenfeld residual plots, shown separately for each endpoint (“AS to AM” or “csPCa”) and biomarker (Proclarix or PSA density).

## Abbreviations

AS	Active surveillance
AM	Active Management
CE	Conformité Européenne
CTSD	Cathepsin D
csPCa	Clinically significant prostate cancer
ciPCa	Clinically insignificant prostate cancer or indolent prostate cancer (iPCa)
DRE	Digital rectal examination
GG	Grade Group
IVD	In Vitro Diagnostic
MRI	Magnetic resonance imaging
NPV	Negative predictive value
PerPros	Personalized Management of Prostate Cancer
PSA	Prostate-specific antigen
PSAD	Prostate-specific antigen density
THBS1	Thrombospondin 1
TRUS	Transrectal ultrasound
