# Supplementary material for: The Prognostic Value of Proclarix in Prostate Cancer Patients Under Active Surveillance: Predicting Transition to Active Treatment and Disease Progression in a Danish Cohort
Source: Cancers (Basel). 2026 Apr 23;18(9):1348. doi: 10.3390/cancers18091348 (PMC13162930; doi:10.3390/cancers18091348)
Supplement: Supplementary file 1 [file cancers-18-01348-s001.zip › cancers-4221810-supplementary.pdf]

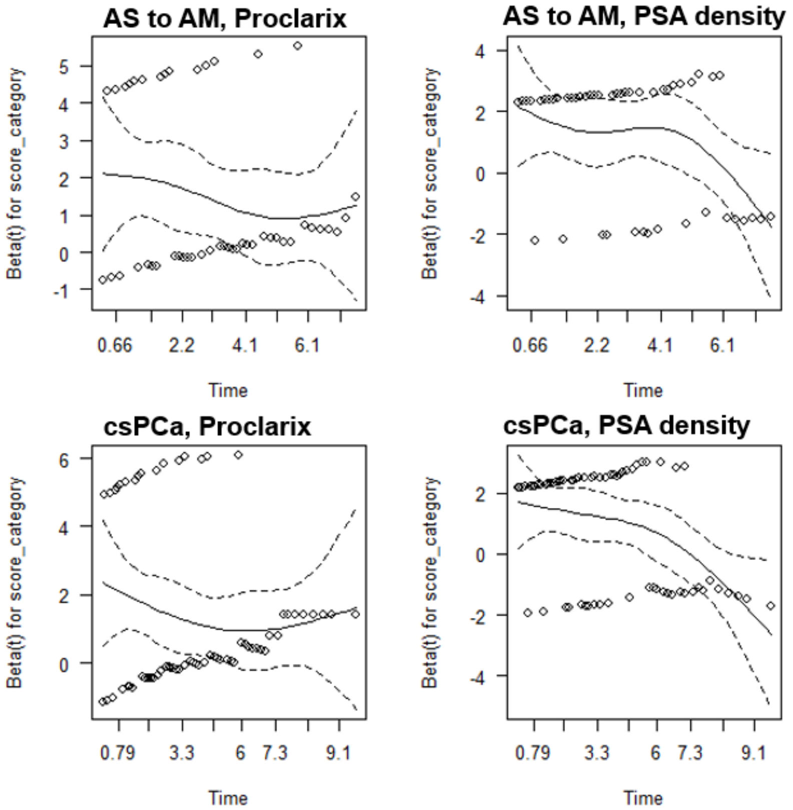

Schoenfeld residuals test p-values for the assessment of the proportional hazard assumption by endpoint, biomarker, and follow-up period

	AS to AM		csPCa	
	**Proclarix**	**PSA density**	**Proclarix**	**PSA density**
5-years follow-up	0.29	0.024	0.59	0.47
Full period follow-up	0.19	0.29	0.15	<0.001

Figure S1: Schoenfeld residual plots, shown separately for each endpoint ("AS to AM" or "csPCa") and biomarker (Proclarix or PSA density). P-values are presented in the table on the right.
